# Characteristics and Outcomes of Left Ventricular-Assist Device-Associated Cerebrovascular Events in Setting of Infectious Intracranial Aneurysms

**DOI:** 10.7759/cureus.15239

**Published:** 2021-05-25

**Authors:** Tanu Garg, Shyam Panchal, Tariq Nisar, David McCane, Jason Lee, Ken Chyuan Ling, Barry Trachtenberg, Arvind Bhimaraj, David Chiu, Rajan Gadhia

**Affiliations:** 1 Vascular Neurology, Houston Methodist Hospital, Houston, USA; 2 Vascular Neurology, Houston Methodist The Woodlands, Houston Methodist Neurological Institute, Houston, USA; 3 Neurology, Houston Methodist Hospital, Houston, USA; 4 Cardiology, Houston Methodist Hospital, Houston, USA; 5 DeBakey Cardiology Associates, Houston Methodist Hospital, Houston, USA; 6 Neurology, Houston Methodist Hospital, Houston Methodist Neurological Institute, Houston, USA

**Keywords:** infectious intracranial aneurysm, lvad, left ventricular assist device, stroke, bacteremia, outcomes

## Abstract

Background and purpose: The study aims to identify the characteristics and neurological outcomes of the left ventricular-assist device (LVAD)-associated cerebrovascular events (CVE) and infections, particularly in the setting of infectious intracranial aneurysms (IIA).

Methods: A single-center retrospective review of patients having undergone LVAD implantation between 2011 and 2017 was conducted using institutional registries and screened for CVE. Patients with CVE were assessed for concurrent bacteremia; neurovascular imaging was then used to isolate patients with IIA. A review of comorbidities, imaging characteristics, and management were performed to determine predictors of neurological outcomes, as defined by the 90-day modified Rankin scale (mRS) scores.

Results: Of the 383 HeartMate II LVAD implantations performed, 43 all-cause stroke events were identified across 35 (9%) patients. The majority of the events were hemorrhagic CVE (n=28) with 21 events complicated by bacteremia. Of patients with hemorrhagic CVE and bacteremia, *Staphylococcus aureus* (n=10) and *Pseudomonas aeruginosa* (n= 8) infection were the most frequently associated organisms. Severe disability or death (90-day mRS > 4) was observed in 15 patients (63%). Seven patients had confirmed findings of IIA on diagnostic cerebral angiogram and were associated with distal middle cerebral artery (MCA) territory involvement (n=6; 86%) with concurrent Staphylococcus (n=5, 71%) and/or Pseudomonas (n=4, 57%) infections. Overall, a higher incidence of acute and chronic bacteremia was observed in the hemorrhagic CVE subgroup compared to the ischemic CVE subgroup (74% vs 36% & 71% vs 29%, respectively; p <0.05). Despite endovascular and/or surgical intervention in patients with IIA, four patients failed management and elected for comfort measures.

Conclusion: Our results indicate that *P. aeruginosa* and *S. aureus* bacteremia are associated with a greater incidence of intracranial hemorrhage and worse neurological outcomes. Future management considerations may include pre-implantation cerebrovascular imaging to assess vascular pathology including prior aneurysms and intracranial atherosclerotic disease burden as a screen for higher-risk patients, as well as more aggressive antibiotic therapy at bacteremia onset.

## Introduction

Left ventricular-assist devices (LVAD) have been established as temporary options as a bridge to heart transplants and as a destination therapy for patients who may not be transplant candidates [1,2]. Cerebrovascular events (CVE) and infection are among the most common complications of LVAD therapy leading to higher rates of morbidity and mortality [3]. It has been reported that the majority of infections occur at the drive-line exit site, and Staphylococcus and Pseudomonas species are the most commonly identified causative organisms [3]. Moreover, there is an increased incidence of CVE in LVAD patients with bacteremia, particularly hemorrhagic CVE (hCVE) over ischemic CVE (iCVE) [1,4,5].

The development of bacteremia in LVAD supported patients has been associated with infectious intracranial aneurysm (IIA) formation [6]. Although the incidence of LVAD-associated IIA is not well defined, higher mortality has been anecdotally reported. Further, there is a paucity of data defining the characteristics and neurological outcomes of LVAD-associated CVEs and infections, particularly in the setting of IIAs. A retrospective observational study was conducted to help identify specific patient characteristics which may be associated with a higher risk of cerebrovascular complications due to IIAs and to suggest preventative measures that could be taken to modify and reduce risk.

## Materials and methods

Patient population

**Figure 1:**
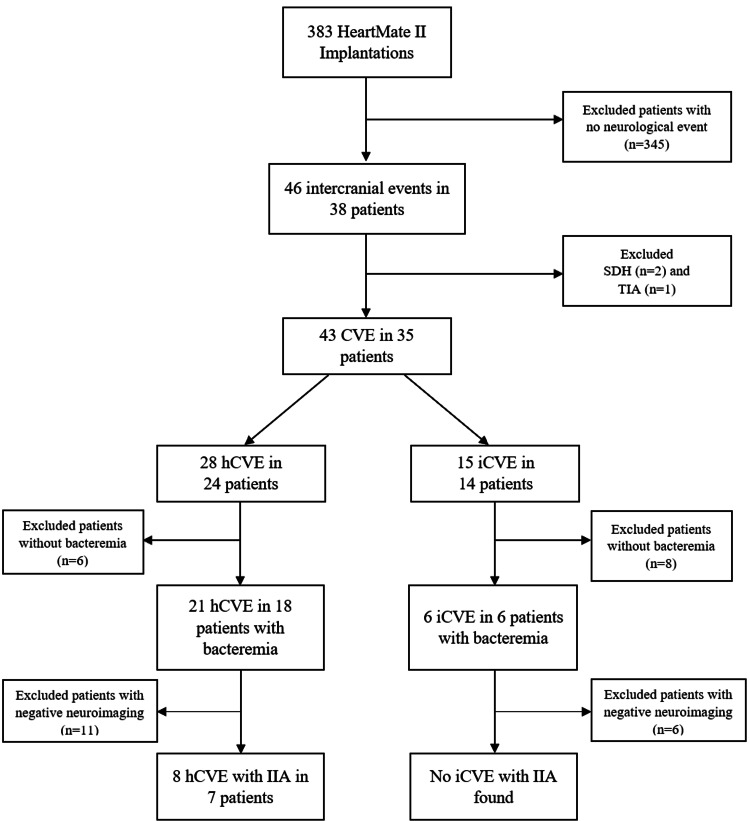
Patient selection process CVE: cerebrovascular event, hCVE: hemorrhagic CVE, iCVE: ischemic CVE, IIA: infectious intracranial aneurysm. No patient with iCVE and bacteremia was found to have IIA.

Electronic charts of consecutive patients who underwent HeartMate II (HM II) LVAD implantation between 2011 and 2017 as a bridge to transplant or destination therapy were reviewed using institutional registries (LVAD and Houston Methodist Hospital Outcomes-based Prospective Endpoints in Stroke, HOPES registry [7]). Figure 1 outlines the patient selection process. Baseline clinical demographics, medical histories, and cerebral imaging data including non-contrast CT brain, CT angiogram of head and neck, and digital subtraction four-vessel cerebral angiograms were obtained from medical records under an approved Institutional Review Board protocol.

Patients on LVAD support were screened for all-cause CVE, which was further divided into hemorrhagic and ischemic stroke subtypes. Patients with hCVE were assessed for concurrent bacteremia; neurovascular imaging including CT angiogram and digital subtraction cerebral angiogram was then used to isolate patients with associated IIA.

We further reviewed comorbidities, imaging characteristics, and management to determine the predictors of neurological outcomes, as defined by the 90-day modified Rankin scale score (mRS). Patients with non-bloodstream infections, transient ischemic attacks, and subdural hematomas were excluded for the purpose of studying the most likely concrete causative ischemic and hemorrhagic events.

Definitions

Bacteremia was determined by means of laboratory testing per standard of care at our center. Acute bacteremia was defined with identification of positive blood cultures on initial presentation upon admission; chronic bacteremia was defined based upon the presence of documented persistent drive-line infections, or positive blood cultures on previous admissions necessitating long-term antibiotic therapy. Bacteremia management was conducted under the guidance of an infectious disease consultant. CVE were defined as a clinical neurologic deficit with correlative neuroimaging findings and were differentiated into hCVE or iCVE. All patients were consulted and evaluated by a board-certified vascular neurologist. hCVE included any intraparenchymal hemorrhage (IPH) and subarachnoid hemorrhage (SAH); extra-axial hemorrhages were excluded from the analysis. Stroke outcomes were defined using the mRS at 90 days from the stroke event. A good clinical outcome was defined with mRS < 4, with poor clinical outcome defined as mRS > 4 (Table 1).

**Table 1:** Neurological disability outcomes

	All CVE (n=35)	hCVE (n=24)	iCVE (n=14)	p-Value
Good clinical outcome (mRS 0-3; n=6)	18.2%	13.6%	27.3%	0.37
Poor clinical outcome (mRS 4-6; n=27)	81.8%	86.4%	72.7%	0.37

Statistical analysis

Statistical analyses were performed using R version 3.6.1 (R Foundation for Statistical Computing, Vienna, Austria). Continuous variables were presented as means ± standard deviation along with medians and quartiles. Categorical variables were presented as percentages. A two-tailed independent t-test and Wilcoxon rank test were used to identify the differences between continuous variables, as appropriate. Kruskal-Wallis test was used to report the median differences of continuous variables. Fisher exact test and chi-square test were used to determine the differences between categorical variables, as appropriate. A univariate logistic regression model was used to identify the differences in binary outcomes. Odds ratio (OR) and 95% confidence intervals (CI) and p-values are reported. A p-value <0.05 was considered significant.

## Results

Of the 383 HeartMate II LVAD implantations performed between 2011 and 2017, 46 all-cause CVEs were identified among 38 patients. After excluding patients with subdural hematoma (n=2) and transient ischemic attack (n=1), a total of 43 events were observed in 35 patients. Overall, 15 iCVE events were identified among 14 patients, and 28 hCVE events were identified among 24 patients. Given the potential for a single patient to have multiple events (hCVE or iCVE) and/or subsequent recurrent events (new hCVE or iCVE), the observed stroke events are greater than the total number of patients.

Baseline characteristics are listed in Table 1. The mean age of all-cause stroke patients was 58 + 10.5 years, with a non-significant trend towards older age for iCVE. No significant differences were found between the two groups as it related to gender, ethnicity, or medical comorbidities. Most patients were managed on a regimen of warfarin and aspirin (58%) or warfarin alone (31%). A non-significant trend was observed towards higher INR values in those with hCVE compared to iCVE (2.20 vs 1.70, p=0.47). There was a higher incidence of acute bacteremia (73% vs 35%; p<0.001) and chronic bacteremia (70% vs 28 %; p<0.05) in hCVE compared to iCVE patients. Univariate analysis showed that chronic bacteremia was seven times more likely to occur in hCVE as compared to iCVE (OR = 7.08, 95% CI [1.60 - 31.33]) as depicted in Table 2. There was a non-significant trend towards worse neurological outcomes (mRS > 4) among patients with hCVE. In patients with more than one hemorrhagic or ischemic event, the highest 90-day mRS recorded was used for analysis as the investigators felt it best reflected the patient's neurological outcome.

**Table 2:** Baseline characteristics INR: International Normalized Ratio, CVE: cerebrovascular event, hCVE: hemorrhagic CVE, iCVE: ischemic CVE. *Other regimens observed included: (1) warfarin + aspirin + other antiplatelets, (2) heparin only, or (3) heparin + aspirin.

Variable	All CVE (n=35)	hCVE (n=24)	iCVE (n=14)	p-Value
Age, years (mean ± SD)	58.10 ± 10.5	55.80 ± 10.8	62.10 ± 9.1	0.06
Gender				
Male (n=29)	76.30%	70.80%	85.70%	
Female (n=9)	23.70%	29.20%	14.30%	0.43
Ethnicity				
White (n=19)	50.00%	45.80%	57.10%	0.73
African American (n=12)	31.60%	29.20%	35.70%	0.72
Hispanic (n=7)	18.40%	25.00%	7.10%	0.22
Comorbidities				
Hypertension (n=37)	97.40%	95.80%	100.00%	1
Hyperlipidemia (n=10)	2.70%	25.00%	30.70%	0.71
Diabetes mellitus II (n=11)	29.70%	25.00%	38.50%	0.46
Chronic kidney disease (n=13)	35.10%	33.30%	38.50%	1
Anti-thrombotic regimen				
Warfarin + aspirin (n=15)	57.70%	40.90%	42.90%	1
Warfarin only (n=18)	31.00%	54.50%	42.90%	0.75
Others* (n=3)	11.50%	4.50%	14.30%	0.54
INR (median and quartile [25% and 75%])	2.0 [1.4–3.1]	2.2 [1.7–3.1]	1.7 [1.4–2.2]	0.47
Bacteremia				
Acute (n=22)	66.70%	73.90%	35.70%	<0.001
Chronic (n=21)	56.80%	70.80%	28.60%	0.01

Fifteen iCVE events were observed in 14 patients. Within this group, one patient was noted to have concurrent convexal SAH, and another was complicated by the hemorrhagic transformation of the ischemic stroke. Another two patients also demonstrated evidence of hypoxic-ischemic injury. These events were classified as iCVE because ischemic infarction was felt to be the primary contributor to the presenting deficits. Bacteremia was identified in six patients, manifesting as acute on chronic bacteremia (n=2), chronic bacteremia with no acute findings (n=2), or acute bacteremia with no prior history (n=2). No patient with iCVE and bacteremia was found to have evidence of IIA. Of note, one patient with iCVE without bacteremia was found to have a small incidental posterior inferior cerebellar artery aneurysm on vascular imaging, believed by the clinician to be non-infectious in etiology. Of the iCVE patients with bacteremia with known 90-day outcomes (n=4), mRS > 4 was seen in three patients; given the high morbidity, all three patients elected for a care plan of comfort measures and withdrawal of aggressive care.

Twenty-eight hCVE events were identified across 24 patients. Most of the observed events were categorized as IPH (n=16), although some were identified as IPH with interventricular extension (IVH; n=3) or IPH and SAH (n=3). One patient presented with a multi-compartmental hemorrhage.

Twenty-one hCVE events were complicated by bacteremia, the majority of which were manifested as acute on chronic bacteremia. Among the 18 patients who had bacteremia, *Staphylococcus aureus* (n=10) and *Pseudomonas aeruginosa* (n= 8) infections were the most frequently identified organisms. Three patients had multiple bacterial infections, and one patient had co-infections of bacterial and fungal organisms. Severe disability or death (90-day mRS > 4) was seen in 15 patients (63%). IIA was identified exclusively in bacteremic hCVE - of all bacteremic hCVE events, eight were attributed to IIA. No patient with iCVE was found to have an associated IIA. Aneurysm characterization was variable and included fusiform, lobulated, and pseudo-aneurysmal features.

Seven patients had confirmed findings of IIA on a diagnostic cerebral angiogram. These were typically associated with distal middle cerebral artery (MCA) territory involvement (n=6, 86%) and concurrent Staphylococcus (n=5, 71%) and/or Pseudomonas (n=4, 57%,) infections. As shown, one patient suffered recurrent hCVE associated with separate IIAs. All patients underwent medical management of hemorrhages including coagulopathy reversal and blood pressure control as indicated by the admitting cardiologist and neurology consultant, and three underwent surgical intervention and hematoma evacuation. Aneurysm management was largely through endovascular embolization; however, two patients did require further micro-surgical resection.

Despite the intervention, four patients failed management post-operatively and had care withdrawn in accordance with patient and family wishes. Ultimately, all seven hCVE patients with IIA had poor functional outcomes at 90 days (mRS > 4), and none survived one-year post-CVE.

## Discussion

In this retrospective single-center study, chronic bacteremia was demonstrated to be a significant risk factor for hCVE. Furthermore, patients with hCVE attributable to IIA suffered very poor neurologic morbidity and mortality outcomes despite intervention, illustrating the severe combined effects of bacteremia and stroke in LVAD-supported patients. To the best of our knowledge, this study represents the largest single-center group of patients with LVAD-associated CVE and IIA reported to date.

Infection is a common cause of morbidity and mortality among LVAD-supported patients. Three previous studies have shown that persistent bloodstream infections (BSI) may increase the risk of all-cause stroke by sevenfold, with the most common source being driveline infections (57%); *S. aureus* and *P. aeruginosa* are found to be the most frequent organisms cultured [1]. Importantly, treatment and complete eradication of such infections can be difficult, with severely complicated cases requiring device exchange. This study confirms previous findings implicating *S. aureus* and *P. aeruginosa* as causal organisms in disease processes. Furthermore, this study demonstrates bacteremia remained persistent without clearance despite all patients with hCVE and IIA having been managed on long-term antibiotic therapy. CVE also contribute to a majority of complications associated in patients supported with LVAD, with up to 11% incidence at one year) [1]. They are associated with high morbidity and frequently lead to not only ineligibility for transplant but also higher mortality [8]. The exact pathophysiology of stroke in this population is not understood, however, proposed mechanisms include thromboembolism from a distant site such as the ventricle, the LVAD itself, or the aortic root. This is thought to lead to a decline in cerebrovascular endothelial function and structure with the continuous flow LVADs and LVAD-related infections [1,8].

A recent systematic review and meta-analysis highlight an association between BSI and stroke in LVAD supported patients. Four out of the six studies presented in the review also suggested an association between bloodstream infection and increased incidence of hemorrhagic CVA (RR 5.28, 95% CI 2.65-10.53) with minimal heterogeneity (I2=30%) [9]. Our study not only supports these findings but also complements them by reporting a higher incidence of IIA in the concurrent bacteremia and hCVE patient cohort.

The pathophysiology between BSI and CVE is under speculation, but hypotheses include some association between cerebral microhemorrhage and driveline infections; an increased risk of thrombotic microangiopathy with an infection predisposing patients to hemorrhage similar to what is seen in infective endocarditis has also been suggested [10-12].

IIA constitutes 2.5% to 6.5% of all intracranial aneurysms and is associated with high mortality rates [13,14]. They arise from microbial infection of a normal cerebral arterial wall or a pre-existing aneurysm, resulting in infiltration of both tunica media and adventitia of the vessel wall, proliferation of the intima, and destruction of the internal elastic lamina; hydrostatic pulsation and thrust against the infected wall can lead to further aneurysmal development and growth [13,15,16]. IIA is more commonly described as a complication of infective endocarditis and tends to be clinically silent unless there is a concomitant embolic infarction or aneurysmal rupture with intraparenchymal or SAH [6]. There is a paucity of IIA-associated CVE in LVAD-supported patients reported in the literature and has been primarily limited to observational reports and studies. Of cases reported thus far, organisms associated with known cases of ruptured IIA in LVAD patients associated with infection include *P. aeruginosa*, *Klebsiella rhinoscleromatis,* and *Staphylococcus epidermidis* [6,17,18]. As shown in Table 3, this review demonstrates a high incidence of *S. aureus* and *P. aeruginosa* in IIA-associated CVE and may suggest a higher degree of virulence associated with these organisms. In addition, a majority of cases previously described are isolated to SAH only without IPH, raising the suggestion of distinction compared to other aneurysmal hemorrhages [6,18,19]. However, findings in this study provide evidence of all compartment hemorrhages, including those outsides of subarachnoid spaces. This highlights that IIA, like other non-infectious cerebral aneurysms, can be associated with both SAH and IPH.

**Table 3:** Characteristics of individual patients with hemorrhagic stroke and infectious intracranial aneurysms M: male, F: female, DM: diabetes mellitus, CVE: cerebrovascular events, HTN: hypertension, INR: international normalized ratio, SAH: subarachnoid hemorrhage, IPH: intraparenchymal hemorrhage, MSSA: methicillin-sensitive *S. aureus*, MRSA: methicillin-resistant *S. aureus*, MCA: middle cerebral artery.

	Patient 1	Patient 2	Patient 2.2	Patient 3	Patient 4	Patient 5	Patient 6	Patient 7
Age/gender	69/M	39/M	39/M	32/F	58/M	45/M	46/M	48/F
INR	3.2	3.9	1.8	3.8	1.9	1.3	3.8	1.8
Antithrombotics	Warfarin + aspirin	Warfarin + aspirin	Warfarin + aspirin	Warfarin + aspirin	Warfarin + aspirin	Warfarin + aspirin	Warfarin	Warfarin
Hemorrhage type	SAH	IPH	IPH	IPH + SAH	SAH	IPH	IPH	IPH + SAH
ICH score	--	0	1	0	--	4	0	2
Hematoma expansion	--	Yes	Yes	No	--	No	Yes	Yes
Bleed management	Medical	Medical + surgical (hematoma evacuation)	Medical + surgical (hematoma evacuation)	Medical	Medical	Medical	Medical + surgical (hematoma evacuation)	Medical
Pathogens	Acute: MSSA, *P. aeruginosa*	Acute: MSSA	Acute: MSSA	Acute: MRSA/C. albicans/Serratia	Acute: MSSA	Acute: MSSA, *P. aeruginosa*	Acute: *P. aeruginosa*	Acute: *P. aeruginosa*
	Chronic: MSSA, *P. aeruginosa*	Chronic: MSSA	Chronic: MSSA	Chronic: MRSA	Chronic: MRSA	Chronic: P. aeruginosa	Chronic: P. aeruginosa	Chronic: P. aeruginosa
TTE/TEE	TTE: no thrombus/vegetation	-	TTE: no thrombus/vegetation	-	TTE: no thrombus/vegetation	-	-	TEE: no thrombus/vegetation
Vessel territory	Distal left MCA	Distal right MCA	Distal right MCA	Right PCA, left MCA	Right MCA	Distal right MCA	Distal left PCA	Distal right MCA
Angiogram findings	4 mm fusiform aneurysm	Ruptured 3 mm pseudo	Multiple lobulated/irregular pseudoaneurysms	Fusiform dysplastic aneurysms; L MCA 3 mm; R PCA 3 × 2.5 mm	Small ruptured fusiform + pseudoaneurysms	Multiple fusiform + dysplastic	Fusiform + dysplastic	Complex pseudoaneurysms
Aneurysm management	Onyx embolization	N-BCA and coil embolization; hematoma evacuation; microsurgical resection	N-BCA embolization, hematoma evacuation; microsurgical resection	No intervention	Incomplete onyx embolization, followed by microsurgical resection	Onyx embolization	Onyx embolization; hematoma evacuation	Onyx embolization
Time from CVE to death (days)	319	119	28	62	345	26	152	59
90-day mRS	4	3	6	6	4	6	5	6

In addition to providing a characterization of cerebral hemorrhages, this study provides data to guide the diagnostic assessment of IIAs. While CT angiography is important for initial workup, the distal location, small size, morphological variance, and multiplicity of IIAs demonstrated in this review show the usefulness of digital subtraction angiography (DSA) for definitive diagnosis and management.

There are no controlled trials for the management and treatment of IIAs to date. Strategies are divided into invasive and non-invasive approaches, although are not mutually exclusive of each other. Anti-microbial agents alone with close radiographic monitoring are considered, as well as endovascular, and/or surgical approaches which are based on clinical experience and prior underpowered studies [6,15]. Matsubara et al. reported treatment of seven aneurysms (six unruptured, one ruptured) with medical therapy alone in patients with IIA of all causes; the case series showed resolution and no aneurysm recurrence or rebleeding in the follow-up period. However, the authors comment that if the IIA enlarges, fails to resolve, or ruptures on medical management, the next recommendation is typically for neurosurgical or neuro-endovascular treatment [20]. Regarding surgical intervention, it has been shown that endovascular management is associated with easier access to distal locations, higher success in the treatment of multiple aneurysms and lower hemorrhagic risk compared to microsurgical craniotomies, and can potentially be performed under non-general anesthesia, an important consideration in cardiac patients [20-22]. In terms of embolization material, detachable coils or liquid embolization agents such as N-butyl cyanoacrylate, Onyx, or others have been used [21]. The friability of the vessel walls and wide neck structure can make surgical techniques such as clipping difficult [23]. Aneurysms that are associated with large IPH or mass effect are considered for further surgical intervention if the parent vessel supplies eloquent areas and revascularization is required to spare the distal arteries, or if endovascular intervention is not technically feasible [21,22]. As has been demonstrated in this review, while many patients may remain on long-term anti-microbial regimens indefinitely, some may undergo endovascular and surgical intervention for IIA-associated CVE. Despite endovascular and surgical intervention, the outcomes observed were poor.

There are a number of limitations of this study given its retrospective nature, namely a small sample size. Additionally, we utilized data from a single site evaluating only patients with HeartMate II implants leading to the small sample size. Ninety-day mRS scores were not recorded for eight patient events, which may have impacted the significance of the univariate analysis. Despite a standard definition for bacteremia, the timing of ordering blood cultures and treatment can vary depending on the treating physician; albeit all patients with bacteremia were evaluated by the infectious disease team. Unfortunately given the lack of standard guidelines for cerebrovascular imaging in patients presenting with LVAD infection, the true incidence and prevalence of IIA is not known in this population. Additionally, given the use of HOPES registry data for patient selection and assessment of outcomes, the outcomes of patients with LVAD-associated bacteremia and non-neurological events remain unanswered.

## Conclusions

While the risk of cerebrovascular events in LVAD patients is known, the incidence, characterization, and neurological outcomes of patients with LVAD-associated CVE in the setting of IIA are poorly defined. Our results indicate *P. aeruginosa* and *S. aureus* bacteremia are associated with a greater incidence of hemorrhagic stroke and worse neurological outcomes. Further investigation is warranted to better define management strategies for this cohort of patients. Future considerations may include pre-LVAD implantation cerebrovascular imaging such as CT angiography to assess for vascular pathology including prior aneurysms and intracranial atherosclerotic disease burden as a screen for higher-risk patients and if it should be part of the selection process for LVAD implantation, as well as more aggressive and/or prolonged duration of antibiotic therapy at bacteremia onset in such patients. LVAD supported patients with persistent bacteremia may also be considered for cerebrovascular imaging as an additional screen for high-risk patients irrespective of acute neurological deficits. More robust prospective studies are needed to better study screening tools, timing of completion, and early therapeutic intervention strategies to better care for our LVAD patients.
